# Healthcare Dilemma Towards Gift Giving by Patients

**DOI:** 10.21315/mjms2021.28.5.14

**Published:** 2021-10-26

**Authors:** Yusrita Zolkefli

**Affiliations:** Pengiran Anak Puteri Rashidah Sa’adatul Bolkiah (PAPRSB) Institute of Health Sciences, Universiti Brunei Darussalam, Brunei Darussalam

**Keywords:** gift giving, professionalism, ethics, dilemma, professionals, healthcare

## Abstract

Gift giving is generally well-intentioned and graciously accepted to healthcare professionals but it is also one of the concerns that cause an ethical dilemma in health care. Is the gift giving ethically appropriate? Tangible guidance about how healthcare professionals would respond to gift giving is possibly scarce and non-specific. In cases where there is an absence of hospital policy specifying how to treat patients’ gifts, healthcare professionals may need to reflect several factors when addressing the dilemmas. This factor includes a reflection on the implications of responding to the gifts.

## Introduction

Gift giving from patients to healthcare professionals usually well-intentioned and graciously embraced but it is still one of the grey topics that create ethical uncertainty in the healthcare context. Is the gift giving ethically appropriate? What factors does one need to consider in deciding whether to accept or decline such gifts? In some cases, a clear policy existed in addressing gift giving whereby the emphasis is made on rejecting gifts intended to encourage preferential treatment. This stance is echoed in many opinion pieces (1). For example, the General Medical Council (GMC), United Kingdom (2) states that doctors must not accept patients’ gifts if they can affect or be perceived to affect how they are treated. This is also mirrored in other Code of Conduct guidance that there is a provision not to accept gifts that could undermine healthcare professionals’ integrity, place them in a conflict-of-interest situation or the worst cases, is get them in legal trouble with alleged bribery and corruption. For example, in the Malaysian context, maintaining a workplace culture with strong ethics and integrity is part of a competent governance framework. It is also crucial toward creating a just, transparent, and free from bribery and corruption (3). Moreover, the most substantial reason against acknowledging single patient gifts is the potential clash of values and interests. Geppert (4) explained that while the guidelines about accepting patient gifts are relatively clear-cut, it is the clash of values around patient gifts which often are steered and disarrayed by emotion. Perhaps employing the ‘just say no’ policy approach could be far simpler and safer. However, is that the simple answer to ethical uncertainty? On the other hand, when there is no such policy, uncertainties occur, resulting in serious moral and ethical repercussions in healthcare practice. Among the examples that depict the problems with accepting gifts is British psychiatrist Peter Rowan in 2011, who was reported to have received a £1.2 million legacy and a gift of £150,000 in payments from an older patient. The GMC ruled that such behaviour of receiving gifts from a patient was deemed inappropriate and fell short of the ethical and professional standards of a medical professional. Peter was later removed from the medical register (5).

## Types of Gifts

It is essential to distinguish between gifts of various forms and categories. First are gifts to mark an occasion, which often include a simple and inexpensive gift. This category includes a poem composed by a child and her parents followed by a box of the doctor’s favourite sweets at the end of a rotation in the paediatric oncology ward or a gift card with a photo of the child achieving a critical developmental milestone following an extended stay in the neonatal ward. It would be disrespectful to refuse such a gift. Secondly, gifts that are regarded as appreciative and a symbol of ‘Thanks’. Foods or flowers offered to the ward staff may illustrate this gratitude and refusing them might hurt the patient’s feelings. Thirdly, gifts that are manifestly ‘over the top’ may be refused to align with good ethical practice. Fourthly, gifts viewed as being in the grey domain between categories two and three, which is the crux of this paper.

## Accepting the Gifts

Among the numerous considerations when accepting a gift from a patient is to reflect at the value of the gift in terms of its value. Some maintain that accepting small, modest gifts is benign. However, there appears to be an understanding that accepting large, costly gifts would be unethical and improper technique (6). There have been cases where health professionals accepted large monetary gifts attracted media attention and eroded public confidence (7), as mentioned earlier. It is not always crystal-clear for the healthcare professionals to gauge the appropriateness of a gift, particularly when it is easily regarded as ‘showing a token of appreciation and a degree of gratitude’. The perceived gift category generally differs and is subject to one’s discretion. For example, what would be considered an excessive gift? If it is a monetary gift, a further difficulty is that it could be seen as ‘tips’ or ‘payoffs’ to healthcare professionals (8).

Meanwhile, the social norms that regulate healthcare professional relationships, called professional boundaries, require a different, more reasoned response (9). In the doctor-patient relationship, for example, the essence of the gifts may progress from consumables that could be shared with the care team to expensive gifts for personal use by the doctor. This evolution may have created a slippery slope situation, making it difficult for the healthcare professional to reject gifts after previous acceptances. For example, even if the healthcare professional ultimately decides not to accept future gifts, finding a comfortable and effective way to do that may be challenging. Snyder (10) states that a small donation to a doctor, such as a gesture of gratitude is not ethically problematic. However, a degree of consideration should still be given to the essence of the gift, the possible consequences for the healthcare professional-patient relationship, and the recipient’s likely intentions and objectives.

Another element that requires assessment is the reason for or intent of the gifts, which is sometimes difficult to determine. It could be that the patient views the gift as simple thanks and expression of gratitude. It could be seen as a social act or expectation, in that the gift is offered as a sign of gratitude. Gift timing can also be important; gifts provided after recognisable interventions may be gestures of gratitude, while gifts given during holidays (e.g. Eid festive) may also represent cultural practices (1). Furthermore, local traditions and customs could be very different (9), which should be considered (11). Social standards typically recommend a respectful thank you after receiving a gift.

It is noteworthy to reflect that, gifts given ‘out of the blue’, mainly when the treatment or care is progressing, should be given further scrutiny, as they might suggest that the patient may expect more in future than the standard of care. In other words, is a gift given to ensure preferential treatment? The complex expectations and interpersonal dynamics present in every therapeutic relationship augment a dimension that healthcare professionals may not always recognise. Being aware of the potential for change in the gift acceptance relationship is critical (7). One must consider whether the gifts may fundamentally and potentially affect the healthcare professional’s relationship with the patient. A gift could be deemed free of any obligations by some, while others believe that gifts create a bond between people and change power relationships (12). Such bond and power relationship changes can be problematic in healthcare contexts, as bonds can lead to feelings of ‘owing’ and ‘expecting something in return’ or the inclination to ask for special prerogatives, no matter how small they may be. Acceptance of any gift may implicitly endorse a special consideration of the health professionals and may clash with impartiality in the patient’s care (10).

We must recognise that there is an inherent risk that accepting a gift might influence the relationship between a healthcare professional and patient and the decision-making as well as promoting his or her partiality toward or special treatment of a patient (13). For this reason, healthcare professionals are encouraged to consider any offer of a patient gift judiciously, to avoid any real or perceived conflict of interest, including alleged abuse of trust or power. In other words, gifts may alter the healthcare professional-patient relationships, making it difficult for them to critically address sensitive issues such as non-compliance with prescriptions, sexual history, or drug abuse (14).

In addition, health professionals ought to focus on fairness and equity when coping with all patients. For example, if healthcare professionals accept gifts from patients who can afford them, will they treat patients who cannot afford gifts differently, particularly when there is a likely temptation to favour them? Will they play favourites and spending more time on patients who give gifts? Will they want to bend the rules? Will they be able to stay objective, and not allow any interests to affect the manner they care or treat the patients? To some degree, it would be too sanguine to suggest that the healthcare professional remain impartial.

In most cases, the bond can potentially sacrifice critical judgement or even violate boundaries by demonstrating inappropriate intimacy. Moreover, gifts may cause disruptions in the workplace. It creates an ethical issue when the patient’s gifts are aimed at a specific healthcare professional, suggesting that the patient has become a favourite. Caring for a patient requires contributions from everyone involved, and that is typically achieved through multidisciplinary efforts. Therefore, if gifts are given to a particular staff member, it is unfair to others, but it could also detract from harmony in the workplace.

## Ethical Scrutiny and Honesty

There is no blanket rule or a one size fits all, responding to patients’ gift giving. Ethical scrutiny is key to improving healthcare professionals understanding and developing ethical responses to gifts. Accepting or declining gifts should not shape the relationship between healthcare professionals and patients. Gifts should never affect patient care. This means that each gift may need to be evaluated on a case-by-case basis (15). Having an ethical scrutiny approach reflects healthcare professionals’ sensitiveness and effort to maintain personal and professional integrity professionalism.

If the health professionals feel uncomfortable or are unable to assess the motivation behind the gift, it should be honestly acknowledged and the gifts to be politely declined. They should also document any gifts that are accepted or rejected. If gifts are rejected, an explanation of the rejection may ease the patient’s feelings and maintain the therapeutic alliance (7). On the other hand, if the gift is deemed appropriate, then the healthcare professionals still have the obligations to advise that the gifts will not change the standard of care. Moreover, suppose a healthcare professional’s conscience tells them that a gift is ethically dubious, such as when it is too costly or create a conflict of interest. In that case, they should be open about the conflict, respectfully refuse the gift and clarify to the patient their reason for doing so, while stressing that it is not the patient who is being rejected. If healthcare professionals are uncertain about approaching the case, they may need to discuss it with colleagues and get advice from them. Healthcare professionals must weigh the potential consequences of gifts carefully. They must also avoid any perception or engagement that might trigger the possibility of a conflict of interest. This is echoed by the statement of the GMC (2) whereby “If you receive a gift from a patient or their relative, you should consider the potential damage this could cause to your patient’s trust in you and the public’s trust in the profession.” Meanwhile, in the absence of a hospital policy or guidelines specifying how patients’ gifts are to be treated, healthcare professionals may need to reflect several factors first, such as the motivation behind the gifts, the impact of accepting the gifts after making it clear to the patient that those gifts are not personal and will be shared with others, and the need to provide clear reasons for declining gifts that are considered inappropriate or make the healthcare professional uncomfortable.

## Conclusion

In summary, gift giving by patients is generally accepted by healthcare professionals. However, it is often necessary that any amount of money or gifts must go through the same ethical scrutiny and honesty to avoid any real or perceived engagement of unethical or unprofessional behaviour. In no way does such scrutiny try to reduce or limit the ‘humanity’ in healthcare professionals and patients’ interaction. If gifts are to be accepted, then there is a more robust ethical duty to be transparent by disclosing such gifts to the appropriate authority. Perhaps it is probably far safer to avoid any situations leading to any forms of conflicts of interest by declining the gifts in the first place.
